# Assessment of neutralizing antibody responses after natural SARS-CoV-2 infection and vaccination in congolese individuals

**DOI:** 10.1186/s12879-022-07593-y

**Published:** 2022-07-13

**Authors:** Armel Landry Batchi-Bouyou, Jean Claude Djontu, Jeannhey Christevy Vouvoungui, Claujens Chastel Mfoutou Mapanguy, Line Lobaloba Ingoba, Jiré Séphora Mougany, Kamal Rauchelvy Boumpoutou, Steve Diafouka-kietela, Raoul Ampa, Francine Ntoumi

**Affiliations:** 1 Fondation Congolaise pour la Recherche Médicale (FCRM), Villa D6, Campus OMS, Djoué, Brazzaville, Republic of Congo; 2 Faculty of Sciences and Techniques, University Marien Ngouabi, Brazzaville, Republic of Congo; 3 Institute of Tropical Medicine, University of Tübingen, Tübingen, Germany

**Keywords:** Vaccine, BBIP-CorV, Janssen/Ad26.COV2.S, SARS-CoV-2, Antibodies, Republic of the Congo

## Abstract

**Background:**

Assessing immune responses after vaccination is part of the evaluation package of vaccine effectiveness in the real world. Regarding SARS-CoV-2, neutralizing antibody levels has been shown to be a good indicator of antibody immune response boosting. So far, limited data have been reported from Africa including in Central Africa. The objective of this study was to provide data on anti-S1 spike total IgG and neutralizing antibodies in vaccinated and non-vaccinated including naturally infected Congolese population during B.1.214.1 and B.1.617.2 variant waves.

**Methods:**

Recruited patients were divided into 4 groups: (1) Naturally infected by the B.1.214.1 variant on January 2021 and followed up until September 2021. These patients have been vaccinated at month 07 and then followed up for 2 months post vaccination; (2) Naturally infected by the B.1.617.2 variant from June 2021; (3) unvaccinated SARS-CoV-2 individuals with no history of prior SARS-CoV-2 infection; (4) fully vaccinated individuals with sinopharm/BBIP-CorV or Janssen/Ad26.COV2.S. SARS-CoV-2 was detected by qRT-PCR and sequenced using Next-Generation Sequencing. ELISA method was used for detecting IgG, and neutralizing Antibody against SARS-CoV-2 antigens using commercial neutralizing assay.

**Results:**

Individuals infected by the B.1214.1 variant elicited consistently high IgG titers at 02, 03 and 06 months. Two months post vaccination with BBIP-CorV, participants showed a significant increase by × 2.5 fold (p < 0.0001) of total IgG and X1.5 fold for neutralizing antibody capacity. This study showed that natural infection with B1.617.2 (delta) variant was more immunogenic compared to those being infected with B1.214.2 variant.

We found a significantly higher concentration in anti-SARS-CoV-2 IgG (p < 0.0002) and antibodies neutralization capacity (P < 0.0001) in fully vaccinated compared to unvaccinated participants. Two months post vaccination, individuals who received Janssen/Ad26.COV2.S presented higher (p = 0.01) total IgG to spike protein compared to BBIP-CorV.

**Conclusion:**

Both natural infection and vaccination with BBIP-CorV and Janssen/Ad26.COV2.S induced antibody response in Congolese population. In addition, Janssen/Ad26.COV2.S was more immunogenic than Sinopharm/BBIP-CorV. There is a need to investigate the duration of these antibodies both in previously infected and naive vaccinated Congolese to allow public heath stakeholders to make evidence-based decision on vaccine schedule for the Congolese population.

## Background

The new human viral pathogen, severe acute respiratory syndrome coronavirus-2 (SARS-CoV-2), the causative agent of the coronavirus disease 2019 (COVID-19) pandemic, emerged in China in December 2019. Since then, vaccines against COVID-19 developed at an unprecedented speed are currently rolled out all over the world [1]. So far, even though hundreds of candidate vaccines have been developed worldwide [2], very limited data are reported about vaccine effectiveness in the African population which has reported the lowest number of cases and deaths/Million inhabitants since the beginning of the pandemic [3].

In Republic of the Congo, eligible persons for vaccination against COVID-19 should be 18 years-old or older including those who have been previously infected with SARS-CoV-2. As of December 31, 2021, approximately 10% of people who have been vaccinated in Republic of the Congo were fully vaccinated to an adenovirus vector vaccine (Janssen/Ad26.COV2.S) or an inactivated-virus vaccine BBIBP-CorV/Sinopharm [SITREP 208]. With regard to the low vaccine coverage, high proportion of the population is exposed to natural infection particularly in Brazzaville and Pointe-Noire [4] which are the two main cities responsible for more than 90% of reported cases (SITREP 161).

Vaccines against SARS-CoV-2 have been shown to elicit levels of neutralizing antibodies comparable to those observed in naturally infected persons [5, 6]. The presence of neutralizing antibodies from prior infection was significantly associated with protection against reinfection [7]. However, it is still unclear which is the necessary titer of neutralizing antibodies that correlate with protection and how long neutralization activity persists in individuals in different conditions and geographical zones.

In 2021, the Republic of the Congo has faced three major COVID-19 waves caused by the B.1.214.1 [8] and B.1.617.2 (Delta) variants and vaccines were introduced in the country in March 2021. This has been an opportunity to enroll and follow up individuals naturally infected and those who received one or two complete doses of COVID-19 vaccine according to the vaccine injection scheme. The available baseline data regarding seroprevalence investigation in RoC dated from March to July 2020 in Brazzaville, the capital [4] and from July 2021 in the North of the country [9]. The reported level of seroprevalence was about 27% in 2020 and between 25 and 67% in 2021. In some African countries, the seroprevalence is growing and expected to reach herd immunity [10, 11]. Thus, reinfection cases [12] show that it is not wise to rely on immunity acquired by natural infection to confer herd immunity.

The main objective of this study was to provide preliminary data on antibody immune responses to SARS-CoV-2 spike protein including total IgG and neutralizing capacity, in well characterized vaccinated and non-vaccinated including naturally infected Congolese population during B.1.214.1 and B1.617.2 variant waves that occurred in the country.

## Methods

### Study design and population

Patients were recruited at the health center of the Congolese Foundation for Medical Research in Massissia, southern district of Brazzaville from January 2021 to November 2021. The criteria for inclusion in the study were (1) to be aged 18 or older; (2) to provide informed consent to participate in the study; (3) to present clinical symptoms suggesting an infection with SARS-CoV-2. For all study participants, socio-demographic data were collected and combined with clinical data of symptoms (headache, fever, etc.). The vaccination status was recorded (the name of the vaccine and dates of vaccination).

Oropharyngeal swabs were collected from each participant for SARS-CoV-2 RT-PCR testing and blood sample for immune responses evaluation.

In the Republic of Congo, vaccination effort started on 24th March 2021. The vaccine coverage was about 1–5% during the study period [SITREP 137, SITREP 161].

The following case definitions were applied to enrolled patients:

1. Naturally infected by the B.1.214.1 variant on January 2021 and followed up until September 2021. These patients have been vaccinated at month 07 and then followed up 2 months post vaccination. Blood and oropharyngeal samples have been collected at 02, 03,06 and 08 months time points after enrolment.
2. Naturally infected by the B.1.617.2 variant from June 2021 (the first case of B.1.617.2 infected individual was mid June 2021 in RoC);
3. Unvaccinated SARS-CoV-2 individuals with no history of prior SARS-CoV-2 infection. As the vaccine hesitancy was high during the study period, many enrolled patients were not vaccinated and based on recruitment criteria, only individuals tested RT-PCR negative at enrolment and who reported that they have never been RT-PCR or antigen positive test for SARS-CoV-2 were included in this group.
4. A vaccinated group was established and only 2 months post-vaccination participants were enrolled in this study including BBIP-CorV (Sinopharm) or Janssen/Ad26.COV2.S (Johnson & Johnson) vaccinees. Those who were RT-PCR positive at inclusion were excluded from the study.

Sequencing data reported in the study period showed that circulating strains were B1.214.1 and Delta (B1.1.617.2) variant.

### SARS-CoV-2 detection

RNA was extracted from swabs using the QIAamp Viral RNA Mini Kit (Qiagen, Hilden, Germany) according to instructions and subjected to RealStar® SARS-CoV-2 real-time PCR targeting the S gene of SARS-CoV-2 (Altona Diagnostics, Hamburg, Germany) by using a high-performance, high-throughput PCR platform (96 well plates) LightCycler® 480 Instrument II (Roche diagnostics, Mannheim, Germany). Amplicons with a Ct < 30 were sequenced using Next-Generation Sequencing (NGS).

### SARS-CoV-2 NGS sequencing

Oxford Nanopore sequencing Technology (ONT) was used. Libraries were prepared as described in Freed et al., (RAPID barcoding, 1200 bp amplicon) [13]. The libraries were quantified (Qubit DNA BR, Thermo Scientific), and sequenced on ONT. The FastQ files obtained from sequencing were analyzed using artic network field bioinformatics [14] pipeline for ONT data**.** Sequences were deposited in GISAID [15] and the lineages of these genomes were annotated by Pangolin online tool [16].

### SARS-CoV-2 specific antibodies detection

#### Measurement of plasma IgG Ab

The IgG Ab against SARS-CoV-2 antigens were measured using GSD NovaLisa® SARSCoV-2 (COVID-19) quantitative IgG (NovaTec Immundiagnostica GmbH) according to the manufacturer’s protocol. Sample preparation included the dilution of plasma with the dilution buffer (1:101). For the assay 100 µL of Negative Control, Positive Control, and each diluted plasma were added to the corresponding wells of micro-plate pre-coated with SARS-CoV-2 antigens, and incubated at 37° C for 30 min. After washing, 100 µL of enzyme substrate, tetramethyl benzidine (TMB), were added to each well, and incubated for 30 min at 37° C in the dark. Finally, 50 µL of stop solution was added to each well and the optical density (OD) for each well immediately measured at 450 nm using an ELISA microplate reader. Quantitative results obtained in Arbitary Unit/ml (AU/ml) were converted to International Units (IU/ml) by multiplying 4.5 in accordance with WHO specifications. If the ratio was above 49.5 IU/ml, it was considered as positive. Negative Control, Positive Control, and Calibrator Control (mix of Positive Control with Negative Control) were included in each assay for quality control.

### Measurement of anti-SARS-CoV-2 neutralizing antibodies

The presence of anti- SARS-CoV-2 Antibodies in the study participants plasma was investigated using cPass™ SARS-CoV-2 Neutralization Antibody Detection Kit (Nanjing GenScript Biotech, China) according to the manufacturer’s protocol [17]. Plasma samples and controls were diluted with sample dilution buffer (1:9), and the peroxydase conjugated Spike protein receptor binding domain (HRP-RBD) diluted with HRP dilution buffer (1:1000). Diluted Positive Control and Negative Controls as well as each of diluted plasma samples were mixed with diluted HRP-RBD at a volume ratio of 1:1 in tubes, and incubated at 37 °C for 30 min to allow the binding of the circulating neutralization antibodies to HRP-RBD. A volume of 100 µL of the Positive Control mixture, the Negative Control mixture, and each plasma sample mixture was then added to the corresponding wells of the capture microplate which was pre-coated with the human receptor of angiotensin 2 converting enzyme (hACE2) protein, and incubated at 37° C for 15 min. After this incubation step, the microplate was washed with 260 µL of wash solution per well four times to remove the circulating neutralization antibodies-HRP-RBD complexes remained in the supernatant. Following a wash cycle, 100 µL of enzyme substrate, tetramethyl benzidine (TMB) were added to each well and the microplate was incubated in dark at 25° C for 15 min. Finally, 50µL of stop solution was added to each well to stop the reaction, and the absorbance of the final solution for each well immediately measured at 450 nm using an ELISA microplate reader. Both negative and positive controls were included in each assay for quality control. A sample was declared positive for neutralizing antibodies if its inhibition was at least 30%.

### Statistical analysis

The data were analyzed using SPSS version 24 (SPSS Inc., Chicago, IL, USA). GraphPad (version 8.0.4) was used to generate the figures. Categorical variables were presented as numbers (%). Continuous variables were expressed as median (interquartile range, IQR) or mean (± standard deviation, SD). Mann–Whitney U-test or Kruskal–Wallis test were used for observing significant difference in distributions between two or more groups. For continuous variables × 2 or Fisher’s exact test were used. Statistical significance was defined as P values of < 0.05.

## Results

A total of 169 participants were recruited and divided in 4 groups (see Material and Methods) as follows: Group 1 (N = 17); Group 2 (N = 52); Group 3 (N = 50); Group 4 (N = 50). The demographic characteristics of the included participants are shown is Table 1.

*1. Follow up of IgG and neutralizing antibodies to spike protein after natural infection and subsequent vaccination with BBIP-CorV*

**Table 1:** Demographic characteristics of all the four (4) groups included in the study

	Group 1 (Infected by B.1.214.1) N = 17	Group 2 (Infected by B.1.617.2) N = 52	Group 3 (Unvaccinated and uninfected) N = 50	Group 4 (Vaccinated and uninfected) N = 50
Age in years (Median with IQR)	42 (36.5; 47.5)	52 (40.7; 61)	38 (27.7; 48.5)	36.5 (26; 53.5)
Gender				
Female, n (%) Male, n (%)	4 (23.5) 13 (76.5)	28 (54) 24 (46)	23 (46) 27 (54)	24 (48) 26 (52)

N: number of participants in group; n: number of participants in subgroup; IQR: interquartile range

A total of 17 participants (group 1) were enrolled in January 2021 according to inclusion criteria and followed up for 8 months. The median age was 42 years old (36.5; 47.5) including 13 males and 4 females for sex ratio (M/F) of 3.3.

**Fig. 1:**
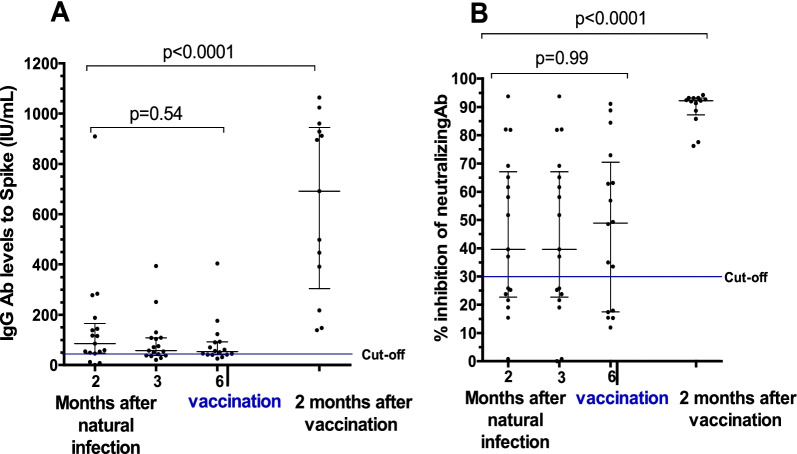
anti-SARS-CoV-2 antibody response to the B.1.214.1 variant two, three and six months after recovery, and 2 months after vaccination. Graph **A** shows the dynamic of IgG concentration. The percentage of inhibition of neutralizing antibodies before and after receiving vaccine is shown in Graph **B**. Kruskal–Wallis-test was used for the comparison

Overall, 70.5% (n = 12/17) of participants elicited stable and high levels of total IgG to the spike corresponding to X2-threefold the minimal threshold (49.5 IU/mL), at 02, 03 and 06 months post natural infection with B.1.214.1 variant (group 1). After two months post vaccination with BBIP-CorV vaccine, a significant increase by × 2.5 fold was observed in all vaccinees (p < 0.001). (Fig. 1A).

With regard to the neutralizing antibodies, 2, 3 and 6 months post natural infection the neutralizing capacity is high and remain also stable over 6 months in 64.7% (n = 11/17) and the vaccination significantly increased by × 1.5 fold the neutralizing capacity of antibodies in the total number of participants in this group (Fig. 1B).

*2. Assessment of antibody response against natural infection with B1.214.1 and B1.617.2 (Delta) variant in Congolese patients*

A total of 69 SARS-CoV-2 RT-PCR positive individuals were enrolled. The infecting SARS-CoV-2 strains were identified in swabs and a blood sample collected two months after acute infection. We recruited 17 and 52 individuals infected with B1.214.1 (group 1) and Delta (group 2) variant respectively**.**

**Fig. 2:**
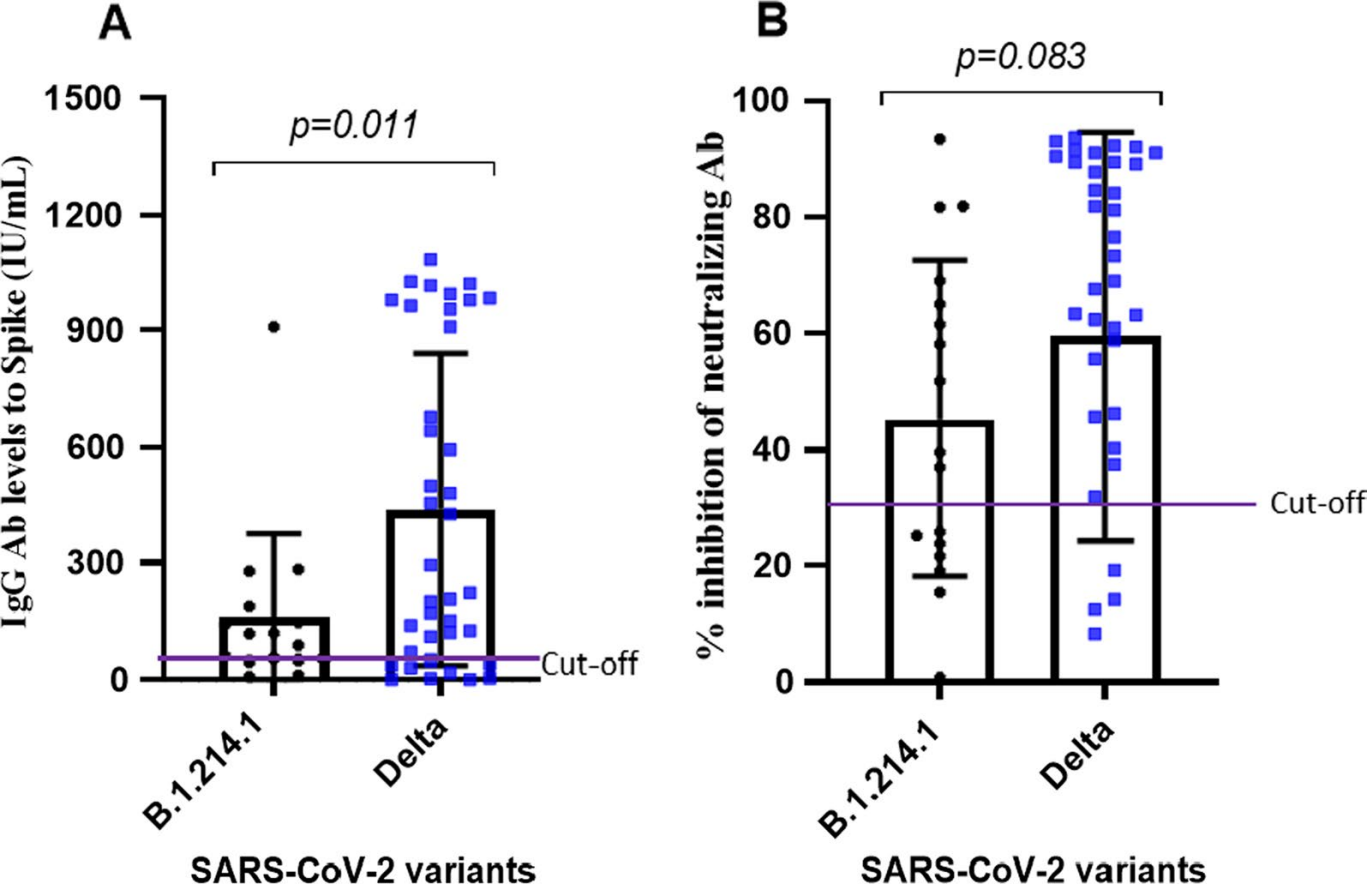
anti**-**SARS-CoV-2 antibody response to both B.1.214.1 and Delta variants 2 months after infection screening. The concentration of the IgG level is found in Graph **A**. The Inhibition percentage of neutralization between B.1.214.1 and Delta variant is shown in Graph **B**. Mann–Whitney Rank Sum test was used for the comparison between two groups

As shown in Fig. 2A, IgG antibodies are significantly (p = 0.011) lower in individuals infected by B1.214.1 compared to those infected with Delta variant two months post-infection. Moreover, a trend to higher % inhibition of neutralizing antibodies was observed in patients who were infected with Delta variant (P = 0.083) (Fig. 2B). The difference in IgG antibody levels was × threefold lower and the inhibition of neutralizing capacity was X 2.5 lower in patients who have been infected by B1.241.1 (respectively Fig. 2A and B). Therefore, infection with Delta variant elicits stronger antibody responses with potent neutralizing capacity.

*3. IgG antibody responses and neutralizing capacity in vaccinated and non-vaccinated Congolese individuals*

**Fig. 3:**
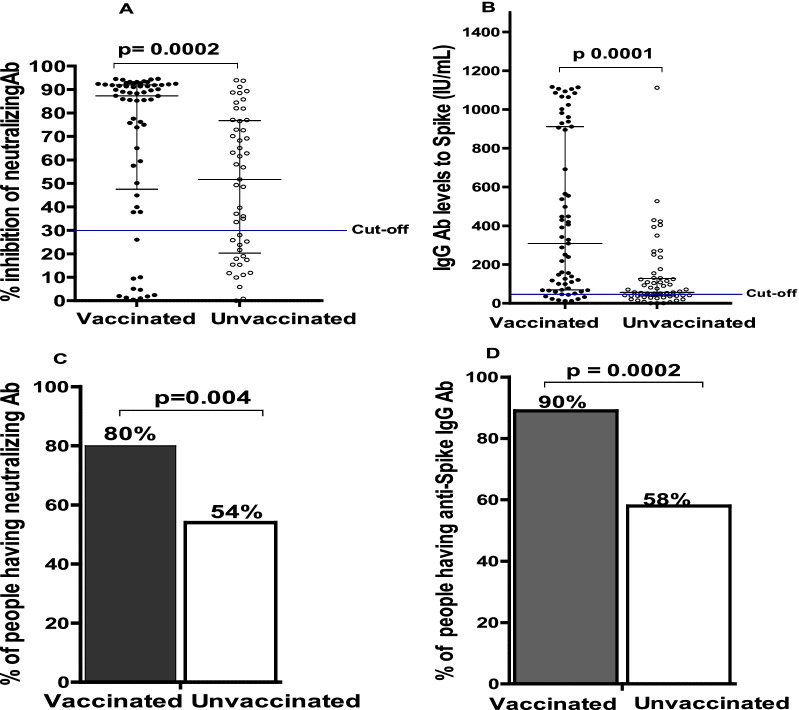
Comparison of antibody responses between vaccinated and unvaccinated. Graph **A** shows the percentage of inhibition of neutralizing antibodies. The concentration of IgG is represented in graph **B**. Graph **C** shows the proportions of people having neutralizing antibodies and graph** D** represents the proportions of participants having IgG antibodies. Mann–Whitney Rank Sum test was used for the comparison of antibodies levels and neutralization capacity, and Fisher exact test for the comparison of percentage of participants with IgG and neutralizing antibodies between two groups

A total of fifty vaccinees (group 4) were enrolled two months post vaccination. They were matched with 50 non vaccinated individuals (group 3). The demographic characteristics of enrolled vaccinees and unvaccinated are presented in Table 1. Twenty-four and twenty-six participants received BBIP-CorV (Sinopharm) and Janssen/Ad26.COV2.S (Johnson & Johnson) vaccine respectively. Figure 3 A and B show a significant difference (p < 0.0002) in % inhibition of neutralization and in IgG to spike (P < 0.0001) between vaccinated and unvaccinated participants.

The proportion of people with neutralizing antibodies is significantly higher in vaccinated participants in 80% of cases (n = 40/50) compared to unvaccinated participants in 54% of cases (n = 27/50) who also reported to have never been infected by SARS-CoV-2 (p = 0.004). Even so, more than 50% of unvaccinated participants had neutralizing antibodies against SARS-CoV-2 spike protein (Fig. 3C, D). Similarly,, 90% (n = 45/50) of vaccinees harbored IgG versus 58% (n = 29/50) in the unvaccinated group (p = 0.0002).

*4. Comparison between vaccine-induced antibody response from BBIP-CorV and Janssen/Ad26.COV2.S*

The two groups of individuals who received BBIP-CorV and Janssen/Ad26.COV2.S vaccines were quite homogeneous in term of gender and age (Table 2). The number of participants with IgG above the threshold is similar (79% vs 88% for BBIP-CorV versus Janssen/Ad26.COV2.S) in both groups of vaccinees. A significant difference (p < 0.014) in the median concentration of IgG was observed (Table 2).

**Table 2:** Demographic characteristics of Congolese participants immunized with SARS-CoV-2 vaccines BBIP-CorV or Ad26.COV2.S

Variables	Vaccinated			Unvaccinated N = 50
	BBIP-CorV N = 24	Janssen/Ad26.COV2.S N = 26	P-value	
Age (years) (IQR)	45 (34.25; 56)	34.50 (26; 49.75)		38 (27.75; 47)
Gender n (%)				
Female	10(41.7)	14(63.9)	0.412	22 (44)
Male	14(58.3)	12(46.1)		28 (56)
IgG results n (%)				
Negative	5(20.8)	3(11.6)		29 (58)
Positive	19(79.2)	23(88.4)	0.456	21 (42)
Median IgG concentration (UI/ml) (IQR)	115 (50.14; 318.1)	550.9 (74.31; 1085)	0.014	58.81 (38.07; 160.1)
Neutralizing antibody results n (%)				
Negative	7(29.2)	6(23)	0.750	23 (46)
Positive	17(70.8)	20(77)	0.431	27 (54)
Median %inhibition (IQR)	62.23 (14.01; 91.51)	87.81 (30.65; 91.14)	0.912	30.75 (4.61; 73.82)

N: number of participants in group; n: number of participants in subgroup; Mean was calculated with standard deviation (SD) and other with a 95% confidence interval (95%.CI); IQR: interquartile range

**Fig. 4:**
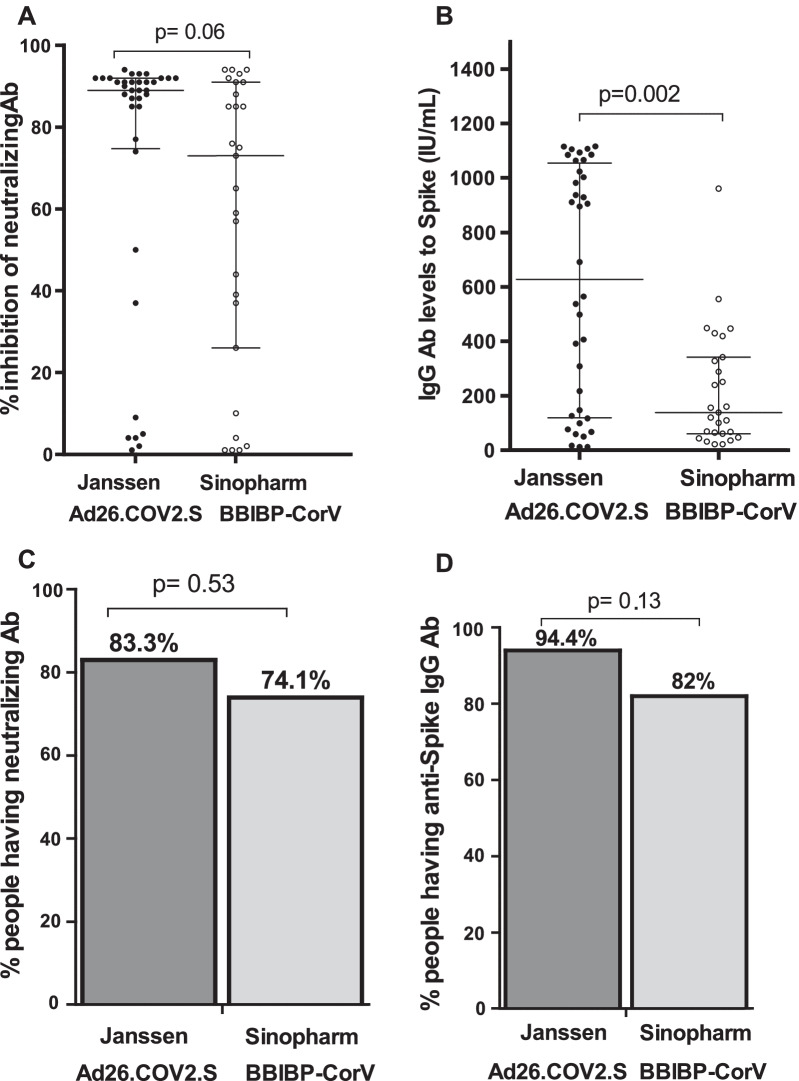
Comparison of antibody responses between Sinopharm BBIBP-CorV vaccine and Janssen/Ad26.COV2.S. Graph **A** shows the percentage of inhibition of neutralizing antibodies. The concentration of IgG is represented in graph **B**. Graph **C** shows the proportions of people having neutralizing antibodies and graph **D** represents the proportions of participants having IgG antibodies. Mann–Whitney Rank Sum test was used for the comparison of antibodies levels and neutralization capacity, and Fisher exact test for the comparison of percentage participants with IgG and neutralizing antibodies between two groups

Two months post vaccination showed a significant difference in total IgG (P = 0.002) (Fig. 4B) in Janssen/Ad26.COV2.S group compared to BBIP-CorV vaccine group. Similarly, neutralization antibody response was slightly higher in Janssen/Ad26.COV2.S group compared to BBIP-CorV vaccine (p = 0.06).

The number of vaccinated participants harboring IgG to spike protein was not significantly higher in Janssen/Ad26.COV2.S group (94%) vs 82% for BBIP-CorV vaccine group. The number of vaccinees with antibodies that had the capacity to neutralize SARS-CoV-2 was also not significantly higher for Janssen/Ad26.COV2.S group (83% vs 74%) (Fig. 4).

## Discussion

Vaccination has a pivotal role for infectious diseases pandemic eradication. Although, COVID-19 cases continue to increase as variants surge, we are experiencing in Sub-Saharan Africa a stagnation of vaccination acceptance rate [18]. In the Republic of the Congo, the vaccination coverage is as low as 10% after one year of vaccine rollout [SITREP 215, 25 January 2022]. Despite the laudable efforts of governments to acquire vaccines through donation or purchase for a targeting coverage of 60% of the population, the key question that is being asked not only by the local stakeholders but also the population is how effective they are in the African population considering the different genetic background as well as the environmental context. The negative attitudes and perceptions regarding COVID-19 vaccines in Central Africa might be attributed to limited evidence of their effectiveness, making it imperative to conduct the current study.

We aimed to assess the neutralizing capacity induced by vaccination and natural infection by using a standardize commercial surrogate virus neutralization test (sVNT) [17]. The sVNT has been designed as a powerful alternative to the conventional plaque reduction neutralization test (PRNT) to measure neutralizing antibodies post-infection to SARS-CoV-2 [19–22], and in vaccinated individuals [23–25]. In addition, the design of the GenScript sVNT ELISA is adapted to determine the difference in binding inhibition to compare vaccines and infecting SARS-CoV-2 variants antibody responses.

Congolese individuals naturally infected by with B.1214.1 SARS-CoV-2 variant harbored high and stable titers of IgG and neutralizing capacity over 6 months, which were boosted significantly by vaccination. Previous published studies conducted in Asia, Europe and USA indicated that S-specific antibodies remained relatively stable post-infection and started waning at six months [19, 26–28]. In contrast to these studies which generally included participants regardless of the SARS-CoV-2 infecting variant, our study focused specifically on antibody kinetics after infection with the B.1.214.1 variant. The B.1.214.1 variant linked to the parent lineage B.1.214 [8, 29] was predominant during the early phases of the pandemic in Republic of the Congo and possibly in other neighboring countries with limited genomic surveillance [30]. The longitudinal study showed that all patients infected by B.1.124.1 variant were tested negative at each checkpoint follow-up, suggesting that the SARS-CoV-2 antibody response induced would have protected them from reinfections by emerging variants.

Consistent with previous observations [31], the generation and maintenance of neutralizing antibodies against SARS-CoV-2 play an important role in resisting reinfections and could be an important indicator for protection [7, 32]. This means that the combined effects of waning antibody responses and increased risk of reinfections suggest that vaccination might be needed to potentialize protection. Our analysis suggests that maximizing neutralizing antibody responses through booster vaccination of previously infected individuals, should be an effective strategy to broadly increase neutralization titers against SARS-CoV-2 variants [33]. In addition, vaccination of previously infected individuals occurred around 6 months after infection. Most boosting studies in previously infected individuals suggested there is a benefit in delaying to 6 months [34]. If fact, 6 months interval is an optimal timeframe enabling an increase in the number of memory B cells after infection responsible of better antibody responses after boosting [35].

Similar to many investigations that evaluated IgG antibody levels after vaccination [36–38], here the groups of naive participants have been followed up two months after vaccination and compared to matched naïve unvaccinated participants. Unsurprisingly, the unvaccinated group showed significantly lower total IgG and neutralizing capacity compared to vaccinated (whatever the used vaccine). This result is important because it shows that in the general Congolese population exposed to SARS-CoV-2 virus and not tested, need to be immunized through vaccination for efficient boosting of their immunity to COVID-19. To date, this finding is the first report showing the importance of vaccination in the Congolese environment and this local evidence is very important to respond to vaccine hesitancy.

When comparing the antibody response from delta variant with that from B.1.214.1, higher IgG levels and neutralizing antibodies were observed with the Delta variant compared to the B.1.214.1 variant 2 months after infection. This may be explained by the fact that the delta variant contains mutations within the spike protein including in the sites specific to neutralizing antibodies, that might lead to more immunogenic epitopes [39–41]. Furthermore, the high viral load caused by the Delta variant which has been reported to be ten times higher than historical SARS-CoV-2 variants, resulting in the production of high levels of neutralizing antibodies needed to inhibit the RBD-ACE2 complex [42].

To our knowledge, this study is the first to report immunological data on BBIBP-CorV vaccine in the African population. As BBIBP-CorV vaccines are largely deployed on the African continent, this information is crucial for national stakeholders as it is difficult to implement vaccination strategies in Africa based on data from Europe and the United States on mRNA vaccines [43–45].

Using the fact that Janssen/Ad26.COV2.S and BBIBP-CorV vaccines were rolled out in the republic of Congo, this study compared immunological responses to both vaccines. It was found that Janssen/Ad26.COV2.S, adenovirus based vaccine[46] induced higher antibody response than BBIP-CorV which is an inactivated virus vaccine. Several lines of evidence have raised questions concerning the structure of S in the inactivated vaccines, suggesting that the combination of purification processes and inactivation with the beta-propiolactone might influence the quality of killed whole-virus vaccines and thus reducing their efficacy [47–49]. In contrast, adenovirus vector vaccines have been reported to induce a robust and long-lasting humoral and cellular immunity [50]. This preliminary result is very interesting and raises the issue of duration of these IgG and neutralizing antibodies in both vaccinated groups. It would be important to investigate further duration of these antibodies in a larger group of individuals. However, the limited (N = 50) number of participants have been addressed by using a test negative design study.

## Conclusions

Both natural infection and vaccination by BBIP-CorV and Janssen/Ad26.COV2.S induced antibody response in Congolese population. Here we found that adenovirus-based vaccine was more able to induce higher total IgG and antibodies neutralizing capacity compared to inactivated virus vaccine. There is a need to investigate now the duration of these antibodies both in previously infected and naive vaccinated Congolese recipients to allow public heath stakeholders to decide on vaccine schedule in the Congolese population.

## Data Availability

The datasets generated and/or analyzed during the current study are not publicly available due to terms and conditions defined by the local Ethical committee, but are available from the corresponding author on reasonable request.

## Abbreviations

ACE2: Angiotensin-converting enzyme 2
ELISA: Enzyme-Linked Immuno Assay
GISAID: Global Initiative on Sharing Avian Influenza Data
HRP: Horseradish peroxidase
IgG: Immunoglobulin G
NGS: Next-generation-sequencing
ONT: Oxford Nanopore sequencing Technology
OD: Optic density
RBD: Receptor-binding domain
RT-PCR: Reverse transcription polymerase chain reaction
SARS-CoV-2: Severe acute respiratory syndrome coronavirus 2
SITREP: Situation report
TMB: Tetramethyl benzidine
WHO: World Health Organization
